# The CoLaus study: a population-based study to investigate the epidemiology and genetic determinants of cardiovascular risk factors and metabolic syndrome

**DOI:** 10.1186/1471-2261-8-6

**Published:** 2008-03-17

**Authors:** Mathieu Firmann, Vladimir Mayor, Pedro Marques Vidal, Murielle Bochud, Alain Pécoud, Daniel Hayoz, Fred Paccaud, Martin Preisig, Kijoung S Song, Xin Yuan, Theodore M Danoff, Heide A Stirnadel, Dawn Waterworth, Vincent Mooser, Gérard Waeber, Peter Vollenweider

**Affiliations:** 1 Department of Medicine, Internal Medicine, CHUV, Lausanne, Switzerland; 2 Institute of Social and Preventive Medicine (IUMSP), University of Lausanne, Switzerland; 3 Outpatient Clinic, University of Lausanne, Switzerland; 4 Department of Medicine, Angiology, CHUV, Lausanne, Switzerland; 5 Department of Psychiatry, CHUV, Lausanne, Switzerland; 6 Medical Genetics/Clinical Pharmacology and Discovery Medicine, GlaxoSmithKline, Philadelphia PA, USA; 7 Center of Excellence for Drug Discovery CV, GlaxoSmithKline, Philadelphia PA, USA; 8 Worldwide Epidemiology, GlaxoSmithKline, Harlow, UK

## Abstract

**Background:**

Cardiovascular diseases and their associated risk factors remain the main cause of mortality in western societies. In order to assess the prevalence of cardiovascular risk factors (CVRFs) in the Caucasian population of Lausanne, Switzerland, we conducted a population-based study (Colaus Study). A secondary aim of the CoLaus study will be to determine new genetic determinants associated with CVRFs.

**Methods:**

Single-center, cross-sectional study including a random sample of 6,188 extensively phenotyped Caucasian subjects (3,251 women and 2,937 men) aged 35 to 75 years living in Lausanne, and genotyped using the 500 K Affymetrix chip technology.

**Results:**

Obesity (body mass index ≥ 30 kg/m^2^), smoking, hypertension (blood pressure ≥ 140/90 mmHg and/or treatment), dyslipidemia (high LDL-cholesterol and/or low HDL-cholesterol and/or high triglyceride levels) and diabetes (fasting plasma glucose ≥ 7 mmol/l and/or treatment) were present in 947 (15.7%), 1673 (27.0%), 2268 (36.7%), 2113 (34.2%) and 407 (6.6%) of the participants, respectively, and the prevalence was higher in men than in women. In both genders, the prevalence of obesity, hypertension and diabetes increased with age.

**Conclusion:**

The prevalence of major CVRFs is high in the Lausanne population in particular in men. We anticipate that given its size, the depth of the phenotypic analysis and the availability of dense genome-wide genetic data, the CoLaus Study will be a unique resource to investigate not only the epidemiology of isolated, or aggregated CVRFs like the metabolic syndrome, but can also serve as a discovery set, as well as replication set, to identify novel genes associated with these conditions.

## Background

Cardiovascular diseases (CVD) are the major cause of early mortality and morbidity in industrialized countries [1]. The prevalence of classical cardiovascular (CV) risk factors (CVRFs) such as hypertension, dyslipidemia, obesity and diabetes varies widely between different countries, and shows some important secular trends

Hypertension, obesity, dyslipidemia and diabetes mellitus have an important genetic component [2,3]. These conditions, however, are genetically complex, and only a small fraction of these diseases are accounted for by Mendelian forms. The availability of large case-control genome-wide association studies has led to the identification of susceptibility genes for common conditions, with even modest effects. Population-based studies have additional advantages; they make it possible to perform association studies for any continuous phenotypic trait which has been properly monitored, as well as for categorical traits using extreme discordant case-control designs, as long as these conditions are sufficiently prevalent. In addition, they offer the opportunity to explore the genetic determinants of aggregated phenotypes such as the metabolic syndrome. Finally, this type of studies provide the opportunity to perform re-sequencing analysis on extremes of the distribution, and to identify rare genetic variants with a strong phenotypic effect [4]. The success of such studies relies on a large collection, detailed and standardized phenotypes, strong analytical capabilities, replication sets and extensive genotyping [5]. To harness the power of these technologies, we designed the CoLaus study. The major goals of the CoLaus Core study were to get a snap picture of the prevalence and severity of CVRFs in a particular population and to elucidate the molecular architecture of isolated CVRFs, as well as clusters of CVRFs like the metabolic syndrome. In this article, we present the protocol of the study as well as results for the prevalence of multiple cardiovascular risk factors the Lausanne population.

## Methods

### Recruitment process and sample size

The Study was approved by the Institutional Ethic's Committee of the University of Lausanne and recruitment took place in the city of Lausanne in Switzerland, a town of 117,161 inhabitants, of which 79,420 are of a Swiss nationality[6].

The complete list of the Lausanne inhabitants aged 35–75 years (n = 56,694 in 2003) was provided by the population register of the city and served to sample the participants to the study. All subjects living in the city of Lausanne in 2003 for more than 90 days have their name included in this register. The register had information on age and gender but no information regarding ethnicity or country of origin.

Given the allele frequency of most SNPs on the Affymetrix chip and the genetic effect sizes expected for complex diseases (i.e. OR 1.3 and above), we chose to include ~6000 individuals, so as to have enough power (~80%) to detect genetic associations for diseases with a prevalence of ~15%.

A simple, non-stratified random selection of 19,830 subjects, corresponding to 35% of the source population, was drawn using STATA v9.1 software (Stata Corp, College Station, USA), and a letter inviting the addressee to participate in the study was sent to these individuals.

Subjects who volunteered to participate were contacted by phone within 14 days by one of the staff members to set up an appointment. Subjects who didn't answer were sent a second invitation letter. If no answer was obtained, they were contacted by phone. Subjects were considered as non-participants if they refused to participate and as non-responders if contact couldn't be made after two successive letters and three successive phone calls. Individuals who didn't live in Lausanne any longer, who were dead or who didn't meet the age criteria were considered as non-eligible. Recruitment began in June 2003 and ended in May 2006.

**Figure 1:**
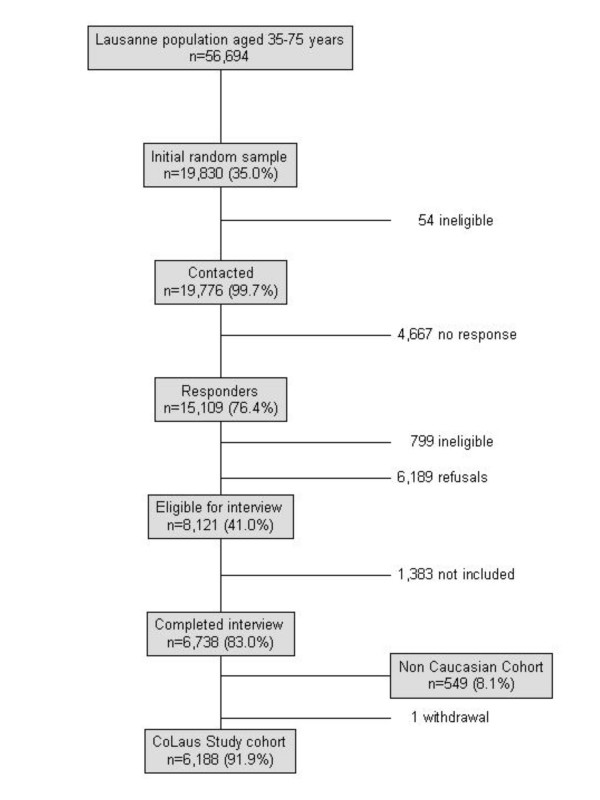
Flow chart of the CoLaus Study.

The sampling procedure is summarized in Figure 1. Of the initial 19,830 subjects sampled, 54 subjects were considered as non-eligible before contact and 15,109 (76%) responses were obtained. A total of 4667 subjects who did not respond were considered as non-responders. Among responders, 6,189 (41%) subjects refused to participate in the study and 799 (5%) were considered as non-eligible. Among the latter, 53% moved to a different city, 32% were out of the age range and 11% were reported to be deceased. The sample of 8,121 subjects who agreed to participate represented 41% of the initially sampled population, 54% of all responders and 57% of all eligible responders.

### Inclusion criteria

The following inclusion criteria were applied: a) written informed consent; b) age 35–75 years and c) Caucasian origin. Caucasian origin was defined as having both parents and grandparents born in a restricted list of countries (available from the authors). No other exclusion criteria were applied. Since ethnicity could only be established during the clinic visit, some participants were assessed but were not included in the CoLaus study. Their data will be described elsewhere.

### Assessment process

Participants were asked to attend the outpatient clinic at the Centre Hospitalier Universitaire Vaudois (CHUV) in the morning after an overnight fast. They had to take their medication as usual. Data were collected by trained field interviewers in a single visit lasting about 60 minutes. Informed consent was obtained from participants upon their arrival at the study clinic. The first questionnaire mailed with the appointment's letter and completed by the participant prior to the morning visit was then quickly reviewed and a second questionnaire was applied by interview prior to clinical measurements and blood collection.

### Questionnaire data

The first set of questionnaires recorded information on demographic data, socio-economic and marital status, and several lifestyle factors, namely tobacco, alcohol and caffeine consumption, physical activity and mood. Data on smoking included the previous and current smoking status as well as the amount of tobacco smoked (number of cigarettes, cigarillos, cigars or pipes), age of beginning and end (for former smokers). Similarly, data on alcohol consumption included the past and current drinking status as well as the number of alcoholic beverage units (wine, beer and spirits) consumed over the week preceding the interview. Caffeine consumption was assessed by the number of caffeine-containing beverages consumed per day. Personal history of overweight and/or obesity and birth weight were also collected. Finally, the 12-item General Health Questionnaire (GHQ12) [7] was applied in order to screen for the presence of non-psychotic psychiatric disorders.

The second questionnaire, administered during a face-to-face meeting with the recruiter, focused on personal and family history of disease and CV risk factors. Subjects were asked which disease(s) they or their family had presented. When a positive answer was given, further information regarding age of occurrence and number of family members affected was collected. When appropriate, death of parents was recorded with age and cause of death. Regarding blood pressure (BP) status, subjects indicated if they had been diagnosed with hypertension and subsequently if they had been, or were being treated currently for this condition. BP levels before the beginning of treatment were sought for and the names of the anti-hypertensive drugs that had been prescribed were collected. In case the anti-hypertensive regimen had been modified, the duration and the reason for changing were also recorded. Personal medicines, including prescription and self-prescribed drugs, vitamin and mineral supplements, homeopathy or natural remedies were collected, together with their main indications. In women, further data regarding reproductive and obstetrical history, oral contraception and hormonal replacement therapy was collected. Finally, an additional frailty questionnaire (for subjects aged over 50 years) and the Mini-Mental State Evaluation (MMSE – for subjects aged over 65 years) were further administered [8].

### Clinical data

Body weight and height were measured with participants standing without shoes in light indoor clothes. Body weight was measured in kilograms to the nearest 0.1 kg using a Seca^® ^scale (Hamburg, Germany), which was calibrated regularly. Height was measured to the nearest 5 mm using a Seca^® ^height gauge (Hamburg, Germany). Body mass index (BMI) was defined as weight/height^2^. Obesity was defined as BMI ≥ 30 kg/m^2 ^and overweight as BMI ≥ 25 kg/m^2 ^and < 30 kg/m^2 ^.

BP and heart rate were measured thrice on the left arm, with an appropriately sized cuff, after at least 10 minute rest in the seated position using an Omron^® ^HEM-907 automated oscillometric sphygmomanometer (Matsusaka, Japan) [9]. The average of the last two measurements was used for analyses. Hypertension was defined as a systolic BP (SBP) ≥ 140 mm Hg and/or a diastolic BP (DBP) ≥ 90 mm Hg during the visit and/or presence of anti-hypertensive drug treatment and was considered as known if the subject was aware of this condition.

In addition, waist and hip circumferences were measured as recommended [10] and fat and fat-free mass were assessed by electrical bioimpedance [11] using the Bodystat^® ^1500 analyzer (Isle of Man, British Isles). Finally, baldness and its age of onset were assessed in men using the Hamilton scale [12].

### Sub-studies

In addition, sub-studies designed to assess the psychiatric characteristics of this population as well as functional CV measurements, were nested onto this study, and will be described separately.

### Biological data

Venous blood samples (50 ml) were drawn after an overnight fast, and most clinical chemistry assays were performed by the CHUV Clinical Laboratory on fresh blood samples whereas Pathway Diagnostics (Los Angeles, CA) measured adiponectin, leptin and insulin [See additional file 1]. Additional aliquots were stored at -80°C.

LDL-cholesterol was calculated with the Friedewald formula only if triglycerides <4.6 mmol/l. Low HDL cholesterol level was defined as <1.0 mmol/L; high HDL cholesterol as ≥ 1.6 mmol/L; high LDL cholesterol was defined as ≥ 4.1 mmol/L and high triglyceride level was defined as ≥ 2.2 mmol/L [13]. In our analysis, dyslipidemia was defined as low HDL cholesterol and/or high triglyceride and/or LDL cholesterol ≥ 4.1 mmol/L or ≥ 2.6 mmol/L in presence of self-reported history of myocardial infarction, stroke, coronary artery disease or diabetes.

Diabetes was defined as fasting plasma glucose ≥ 7.0 mmol/L and/or presence of oral hypoglycaemic or insulin treatment. Type 2 diabetes mellitus (T2DM) was defined in case of diabetes without self-reported Type 1 DM. Diabetes was considered as known if the subject was aware of this condition. Impaired fasting glucose (IFG) was defined as fasting plasma glucose between 6.1 and 6.9 mmol/L without anti-diabetic treatment [14].

A urine sample was collected for the assessment of creatinine and albumin and the albumin-to-creatinine ratio was calculated. Microalbuminuria was defined as a value of albumin-to-creatinine ratio above 30 mg/g.

### Genotyping

Nuclear DNA was extracted from whole blood for whole genome scan analysis and genotyping was performed using the Affimetrix 500 K SNP chip, as recommended by the manufacturer. Subjects consented for the genetic data to be used for the study of cardiovascular risk factors, and associated diseases including mood disorders.

### Data management, security and quality control

Data were entered into a secured, internet-based database. The database was designed to confirm the validity of the identification codes, establish the completeness of the information keyed in and to perform basic data checks. All discrepancies were recorded in the case report form kept in a locked room. Each modification of the data was automatically recorded, including the identity of the investigator who made the modification, the date, the old and the new value.

Staff members were trained and certified before being involved actively in the study. Certification included ability to conduct interviews, to perform phlebotomy and to process blood samples, to accurately measure anthropometric and BP levels and to enter data into electronic databases. The accurateness of the data was checked by an external quality control organization (PRN, North Hampshire, United Kingdom).

Finally, the 'Laboratoire Central du CHUV' is ISO 9001 certified and is regularly checked by the "Centre Suisse de Contrôle de Qualité" (CSCQ – Swiss Centre for Quality Control).

### Power estimates and type 1 error rate

Power calculations were done using the program Quanto v1.1 (James Gauderman, University of Southern California, USA) [15]. Power for unmatched case-control studies was estimated using an arbitrary allelic frequency ranging from 0.1 to 0.5, an additive model, a disease prevalence of 50% and a type 1 error rate of 10^-7^, which was used as a correction for multiple testing taking into account 500'000 genetics markers. Curves were drawn for estimated genetic effect sizes (odds ratios) of 1.2 to 1.8. To estimate power in a continuous trait analysis, SBP was used as a continuous outcome for independent subjects, with an additive model and a type 1 error rate of 10^-7^. Curves were drawn for various minor allele frequencies (0.1 to 0.5). To minimize false positive results, the most significant genetic markers will be replicated in independent samples.

### Statistical analysis

Statistical analyses were performed using Stata 9.1 (Stata Corp, College Station, USA). Results were expressed as mean ± standard deviation (SD) or as number of subjects and (percentage). Data for age group 35–75 years and for the canton de Vaud were extracted from the MONICA population surveys and used to assess trends [16]. Age at sampling was used for comparisons between the initial population, the random sample and the CoLaus study population, whereas age at examination was used to describe the CoLaus Study group characteristics. Comparisons were performed using Student's t-test or chi-square test for quantitative and categorical variables, respectively. Statistical significance was assessed for p < 0.05.

### Sponsoring

The Study was sponsored in part by GlaxoSmithKline and all participants were duly informed about this sponsorship and consented for the use of biological samples and data by GlaxoSmithKline and its subsidiaries.

## Results

### Recruitment

A total of 8121 individuals volunteered to participate in the study. Among these subjects, the first 6,738 were invited to attend the clinic and completed the examination. 549 participants (8.1%) were not of Caucasian ethnicity and were excluded from the CoLaus study and 6,189 participants met the inclusion criteria (including ethnicity) and were included in the CoLaus study. As the number of subjects who agreed to participate (8,121) was higher than the number of subjects initially planned for the CoLaus study (6,000), 1,383 could not be included into the study although they were willing to participate. One subject withdrew after consent due to personal reasons. Overall, the final CoLaus sample (n = 6,188) represents 43% of the eligible responders, 41% of all the responders and 31% of the initially sampled population.

The gender and age characteristics of the source population, the initial random sample and the final CoLaus sample are summarized in Table 1. Overall, both the sampled population and the CoLaus study participants were on average one year younger than the base population, due to an under-representation of subjects aged over 65 years while no differences were found for gender distribution. Further, after excluding non-eligible subjects, no differences were found regarding mean age and gender distribution between the subjects included and the random sample (not shown). The CoLaus Study participants were significantly older (51.1 ± 0.1 vs. 50.8 ± 0.1 years, p < 0.005) than the random sample, while no differences were found for gender distribution (not shown). Age at examination of the participants was on average 2 years higher than age at sampling because of the time elapsed between the two procedures. Distribution of the zip codes within the city was comparable between the base population, the random sample and the CoLaus study participants (not shown).

**Table 1:** Distribution of participants by age and sex in the source population, the initial random sample and the CoLaus Study participants.

	Source population (n = 56,694)	Random sample (n = 19,830)	CoLaus Study (n = 6,188)
Women (%)	30,141 (53.4)	10,601 (53.5)	3,251 (52.5)
*P value*		0.79	0.08
Age (years)	52.0 ± 11.6	50.8 ± 11.5	51.1 ± 10.9
*P value*		<0.001	<0.001
Age group			
35–44	18.877 (33.4)	7,265 (36.6)	2,051 (33.1)
45–54	14,614 (25.9)	5,202 (26.2)	1,682 (27.2)
55–64	12.484 (22.1)	4,417 (22.3)	1,657 (26.8)
65–75	10,524 (18.6)	2,946 (14.9)	798 (12.9)
*P value*		<0.001	<0.001

Results are expressed as number of subjects and (percentage) or as mean ± SD. Statistical analysis by Student's t-test or chi-square test compared to the source population

### Socio-economic characteristics of the CoLaus study participants

The CoLaus Study participants' main characteristics are summarized in Table 2. No gender differences were found regarding the percentage of foreigners. Overall, women were more frequently divorced, widowed or single than men and thus were living more often alone or as a monoparental family (41.7% vs. 23.6%, p < 0.01). Women were also less frequently on a full-time job, more prone to receive social help (26.2% vs. 22.2%, p < 0.001) and had a lower educational level. Among subjects aged less than 40 years old, 48.4% of women had a high school/college/university degree versus 47.4% of men (p < 0.01).

**Table 2:** Socio-economic characteristics of the participants in the CoLaus Study.

	Overall (n = 6,188)	Women (n = 3,251)	Men (n = 2,937)	*P value*
Born in Switzerland (%)	3,997 (64.6)	2,129 (65.5)	1,868 (63.6)	0.12
Marital status (%)				
Single	1,022 (16.5)	569 (17.5)	453 (15.4)	
Married	3,635 (58.8)	1,673 (51.5)	1,962 (66.9)	<0.001
Divorced	1,242 (20.1)	761 (23.4)	481 (16.4)	
Widowed	287 (4.6)	248 (7.6)	39 (1.3)	
Education (%)				
Basic	1,287 (20.8)	777 (23.9)	510 (17.4)	
Apprenticeship	2,286 (37.0)	1,170 (36.0)	1,116 (38.0)	<0.001
High school/college	1,470 (23.9)	804 (24.8)	666 (22.7)	
University	1,140 (18.4)	497 (15.3)	643 (21.9)	
Work status (%)				
Full time	3,790 (61.2)	1,694 (52.1)	2,096 (71.4)	<0.001
Other	2,398 (38.8)	1,557 (47.9)	841 (28.6)	

Results are expressed as number of subjects and (percentage). Comparison between gender was performed using chi-square test.

### Clinical characteristics of the CoLaus Study participants

Men had a higher BMI, waist/hip ratio, systolic and DBP levels, but lower body fat percentage, than women. [Table 3]. Men had higher total cholesterol and triglyceride levels, but lower HDL cholesterol, than women. Fasting blood glucose levels, homocystein, apolipoprotein B, insulin, NT-proBNP and uric acid were higher in men than in women [Tables 3 and 4]. Conversely, women presented higher LDL cholesterol particle size, adiponectin, leptin and hsCRP than men.

**Table 3:** Clinical characteristics of the participants in the CoLaus study, by gender.

	Overall (n = 6,188)	Women (n = 3,251)	Men (n = 2,937)	*P value*
Age (years)	53.1 ± 10.8	53.5 ± 10.7	52.6 ± 10.8	<0.001
Waist/hip ratio	0.88 ± 0.08	0.83 ± 0.07	0.93 ± 0.06	<0.001
BMI (kg/m^2^)	25.8 ± 4.6	25.1 ± 4.9	26.6 ± 4.0	<0.001
Body fat (%)	29.3 ± 9.0	34.4 ± 8.2	23.8 ± 6.1	<0.001
Systolic BP (mm Hg)	128 ± 18	125 ± 18	132 ± 17	<0.001
Diastolic BP (mm Hg)	79 ± 11	78 ± 11	81 ± 11	<0.001
Total cholesterol (mmol/L)	5.59 ± 1.04	5.61 ± 1.03	5.56 ± 1.04	<0.05
HDL cholesterol (mmol/L)	1.63 ± 0.44	1.81 ± 0.43	1.44 ± 0.36	<0.001
Triglycerides (mmol/L)	1.40 ± 1.18	1.16 ± 0.66	1.66 ± 1.52	<0.001
LDL cholesterol particle size (nm)	272 ± 4	273 ± 4	271 ± 5	<0.001
Apolipoprotein B (mg/dL)	1.74 ± 1.34	1.69 ± 1.29	1.80 ± 1.38	<0.005
Glucose (mmol/L)	5.55 ± 1.15	5.34 ± 1.02	5.78 ± 1.23	<0.001
Insulin (μU/mL)	8.44 ± 6.3	7.97 ± 5.47	9.62 ± 6.78	<0.001
Adiponectin (μg/mL)	9.94 ± 8.12	12.32 ± 9.33	7.32 ± 5.43	<0.001
Leptin (ng/mL)	13.1 ± 10.7	16.9 ± 11.7	8.65 ± 7.3	<0.001
Homocystein (μmol/L)	10.4 ± 4.4	9.4 ± 3.2	11.4 ± 5.2	<0.001
hsCRP (mg/L)	2.49 ± 3.48	2.65 ± 3.71	2.30 ± 3.21	<0.001
Pro-BNP (ng/L)	682 ± 531	679 ± 519	686 ± 545	0.60

Results are expressed as mean ± SD. BMI: body mass index; HDL: high density lipoprotein, hsCRP: high sensitivity C-reactive protein, BNP: brain natriuretic peptide. Statistical analysis between gender by Student's t-test or chi-square test.

**Table 4:** Clinical characteristics of the participants of the CoLaus study, by gender.

	Overall (n = 6,188)	Women (n = 3,251)	Men (n = 2,937)	*P value*
ASAT (U/L)	29.96 ± 14.04	26.22 ± 9.88	34.10 ± 16.57	<0.001
ALAT (U/L)	27.84 ± 19.50	21.98 ± 14.47	34.32 ± 22.13	<0.001
Alkaline phosphatase (U/L)	63.47 ± 20.72	62.66 ± 20.99	64.38 ± 20.38	<0.001
Gamma-GT (U/L)	33.20 ± 59.04	22.95 ± 25.78	44.57 ± 79.80	<0.001
Calcium (mmol/L)	2.29 ± 0.09	2.28 ± 0.10	2.29 ± 0.09	<0.001
Albumin (g/L)	44.20 ± 2.53	43.73 ± 2.48	44.71 ± 2.48	<0.001
Total protein (g/L)	74.41 ± 4.39	73.93 ± 4.42	74.95 ± 4.30	<0.001
Uric acid (μmol/L)	313.49 ± 84.47	270.56 ± 67.23	361.08 ± 75.69	<0.001
CDT (% of total transferrin)	0.95 ± 0.81	0.80 ± 0.48	1.12 ± 1.02	<0.001

Results are expressed as mean ± SD. ASAT: Aspartate amino-transferase. ALAT: Alanine amino-transferase. Gamma-GT: Gamma-glutaryl-transferase. CDT: Carbohydrate deficient transferrin. Statistical analysis between genders by Student's t-test or chi-square test.

### CVRFs within the CoLaus study

More than one third of the overall sample was overweight, and slightly less than one-sixth was obese; overweight and obesity were also more prevalent in men [Table 5] and increased with age. In men aged 35–44 years, the prevalence of overweight and obesity were 40.7% and 11.4%, respectively, whereas in men aged 65–75 the corresponding figures were 50.8% and 22.7% (p < 0.001). The corresponding figures for women were 21.9% and 9.6%, and 35.8% and 17.5% (p < 0.001), respectively.

**Table 5:** Prevalence of selected cardiovascular risk factors in the participants of the CoLaus study.

	Overall (n = 6,188)	Women (n = 3,251)	Men (n = 2,937)	*P value*
**BMI status **(%)				
Overweight	2265 (36.6)	922 (28.4)	1343 (45.7)	<0.001
Obesity	974 (15.7)	472 (14.5)	502 (17.1)	
**Smoking status **(%)				
Current	1673 (27.0)	813 (25.0)	860 (29.3)	
Former	2034 (32.9)	904 (27.8)	1130 (38.5)	<0.001
Never	2479 (40.1)	1534 (47.2)	945 (32.2)	
**Blood pressure status (%)**				
Hypertension	2268 (36.7)	1004 (30.9)	1264 (43.0)	<0.001
Treated hypertension	1131 (50.1)	537 (53.8)	594 (47.1)	<0.005
Treated to goal	542 (48.0)	271 (50.6)	271 (45.6)	NS
**Lipid status (%)**				
High LDL cholesterol	1263 (20.8)	631 (19.5)	632 (22.2)	<0.001
High triglycerides	773 (12.5)	216 (6.7)	557 (19.0)	<0.001
Low HDL cholesterol	170 (2.8)	35 (1.1)	135 (4.6)	<0.001
High HDL cholesterol	3296 (53.4)	2324 (71.6)	972 (33.2)	<0.001
Dyslipidemia	2113 (34.2)	862 (26.6)	1251 (42.7)	<0.001
Treated dyslipidemia	286 (13.5)	95 (11.0)	191 (15.3)	<0.001
**Glycaemic status (%)**				
Diabetes	407 (6.6)	130 (4.0)	277 (9.5)	<0.001
Known Diabetes	270 (66.3)	89 (68.5)	181 (65.3)	<0.005
Treated Diabetes	260 (96.3)	85 (95.5)	175 (96.7)	<0.01
**Microalbuminuria (%)**	380 (6.3)	173 (5.4)	207 (7.3)	

**Overweight**: BMI ≥ 25 kg/m^2^. **Obesity**: BMI ≥ 30 kg/m^2^. **Hypertension**: Blood pressure ≥ 140/90 mmHg and/or presence of anti-hypertensive drug treatment. **Low HDL cholesterol**: <1 mmol/L; **high HDL cholesterol**: ≥ 1.6 mmol/L; **high LDL cholesterol**: ≥ 4.1 mmol/L and **high triglyceride**: ≥ 2.2 mmol/L. **Dyslipidemia**: low HDL cholesterol and/or high triglyceride and/or LDL cholesterol ≥ 4.1 mmol/L or ≥ 2.6 mmol/L in presence of self-reported myocardial infarction, stroke, coronary artery disease or diabetes. **Diabetes**: fasting plasma glucose ≥ 7 mmol/L and/or presence of oral hypoglycaemic or insulin treatment. **Microalbuminuria **:albumin-to-creatinine ratio > 30 mg/g

Smoking was reporter by 27% of the participants; the prevalence of current smoking was higher in men and tended to decrease with age, from 35.3% among 35–44 year olds to 20.7% among 65–75 year olds in men (p < 0.001), the corresponding figures being 28.1% and 14.7% in women (p < 0.001). Interestingly, in the 45–54 age class, prevalence of smoking was higher in women than in men (30.7% vs. 28.8% (p < 0.001)).

The prevalence of hypertension was 36.7% overall, was higher in men and increased with age: 18.3% and 75.1% in men aged 35–44 and 65–75, respectively (p < 0.001); the corresponding numbers in women were 9.9% and 59.1% (p < 0.001). Among hypertensive subjects, 50.1% were currently taking anti-hypertensive medication. Treatment for hypertension was more frequent in female subjects and increased with age (from 32.2% to 59.4%). Of the treated hypertensive subjects more than half (52.0%) had BP levels ≥ 140/90 mmHg.

High LDL cholesterol, high triglyceride and low HDL cholesterol levels were seen in 20.8%, 12.5% and 2.8% of the subjects, respectively and these conditions were also more prevalent in men. The prevalence of high HDL cholesterol was 53.4% and higher in women than men (71.6% vs. 33.2%). Overall, one third of the sample had dyslipidemia, the prevalence of which was higher in men (42.7%) than in women (26.6%).

Use of statin therapy in subjects with high LDL cholesterol was 5.6%. Prevalence of statin therapy was 69% in subjects after myocardial infarction, 33% in subjects after stroke and 70% in subjects after coronary artery bypass graft. Target level of LDL-cholesterol for secondary prevention, as recommended (< 2.6 mmol/l), was achieved in 28%, 38%, 28% and 33% of the subjects with diabetes, myocardial infarction, stroke and coronary artery bypass graft, respectively.

The overall prevalence of diabetes was 6.6%, and was higher in men. Nine subjects (2.2%) reported to have T1DM. The prevalence of diabetes increased with age, from 2.5% to 17.2% in men aged 35–44 and 65–75, respectively; the corresponding numbers were 1.2% and 9.0% in women with a peak prevalence of 17.2% in men aged > 65. Roughly a third were newly diagnosed diabetics (31.5% for women and 34.7% for men, respectively). The prevalence of IFG in the CoLaus population was 9.8% and was higher in men than in women (14.3% vs. 5.6%).

Treatment for diabetes was present in 73.9% of diabetic subjects but nearly all known diabetics were treated (96.3%). Of the treated diabetic subjects 63.9% had a fasting blood glucose ≥ 7 mmol/L, at the time of their visit.

Finally, 0.7% of women and 2.6% of men reported a personal history of myocardial infarction (p < 0.001); conversely, no gender differences were found regarding personal history of stroke: 1.0% of women vs. 1.3% of men, p = 0.16.

### Power estimates

**Figure 2:**
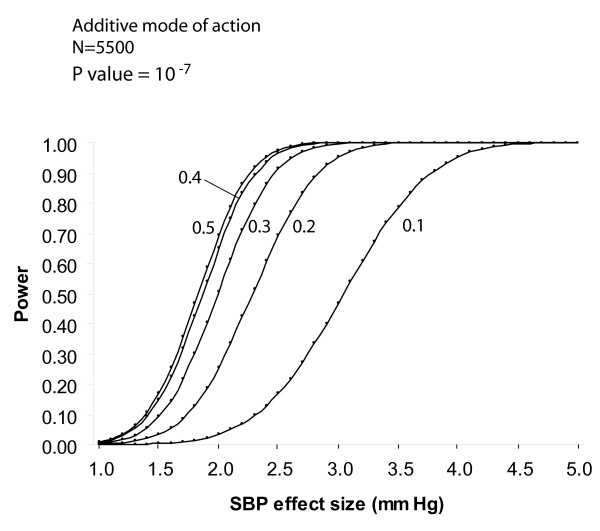
**Power estimates for the CoLaus study. Power for various sample sizes in unmatched case-control studies**. Arbitrary allelic frequency of 0.3, a disease prevalence of 50% and a type 1 error rate of 10^-7^taking into account 500'000 genetics markers. Curves are for estimated genetic effects (odds ratios) of 1.2 to 1.8. The numbers of cases for main CVRFs in the CoLaus study are: Hypertension: 2268. Dyslipidemia: 2021. Obesity: 963. Smoking (> 25 cigarettes/day): 746. Type 2 diabetes: 398. Coronary heart disease: 262. Low HDL: 170.

**Figure 3:**
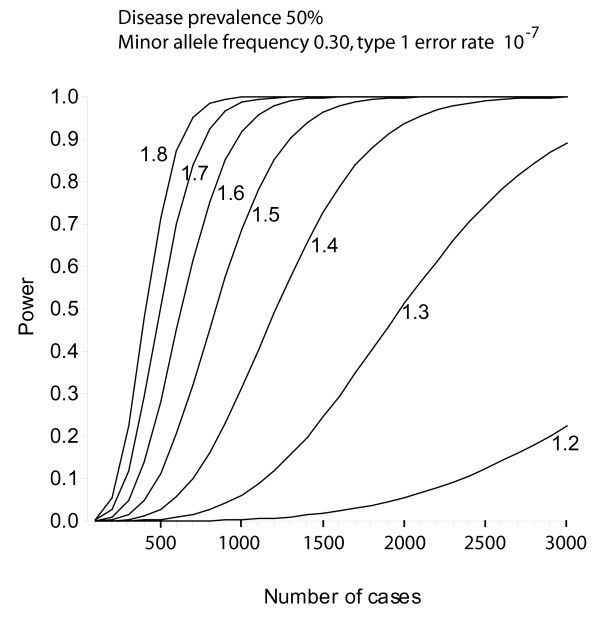
**Power estimates for the CoLaus study. Power for the analysis of systolic blood pressure as a continuous variable**. Power calculations using a continuous outcome for independent subjects, with an additive mode of action for the allele and a type 1 error rate of 10^-7^. Calculations were done for various minor allele frequencies (0.1 to 0.5).

The power of the CoLaus study for an unmatched case-control design to study genotype/phenotype associations depends on the number of cases/controls and estimated effect size [Figure 2]. For hypertension with 2268 cases, the estimated power is 0.9 for a 1.4 effect size and 0.5 for a 1.3 effect size. Cases for main CVRFs in the CoLaus study are: dyslipidemia: 2021, obesity: 974, smoking (> 25 cigarettes/day): 746, Type 2 diabetes: 398, coronary heart disease: 262, low HDL: 170. For continuous trait analysis, the example of SBP was taken [Figure 3]. For allelic frequencies of 0.2 to 0.4, the study has an estimated power of >0.8 to detect BP variations of 2.0 – 2.3 mm Hg.

## Discussion

Described in this report are the rationale, objectives, methods and first results from the CoLaus study, a single-center population-based sample including 6188 extensively phenotyped Caucasian subjects aged 35–75. Our results indicate that the prevalence of major CVRFs is high, in particular in men. In addition, this collection represents a powerful tool to identify new molecular determinants of CVRFs and associated diseases [17].

The participation rate of 41% in the CoLaus study is comparable to the MONICA surveys conducted in Switzerland and in France [18]. The lower response rate among elderly subjects is in agreement with previous data [19] and might be related to a lower interest for the study. However, the distribution of age groups 35–54 and 55–75 in the CoLaus study was comparable to the source population [Table 1]. Also, there was no gender or zip code distribution difference between the source population, the random sample and the CoLaus participants. Although the optimal sampling frame would have consisted of a list of all Caucasians living in the city, information on ethnicity was not available to the investigators before examination and it is not possible to assess whether ethnicity had an effect on the participation rate.

The prevalence of main CVRFs was high in the CoLaus participants. Roughly over half of the participants presented with overweight and obesity, over a third had high BP or dyslipidemia while one in 15 participants had diabetes.

In agreement with the literature [20], men had a higher prevalence of obesity and overweight than women. Further, comparison with data from the MONICA study suggests that the prevalence of obesity is increasing, which confirms what has been found in the nearby city of Geneva [21]. Compared to data from the National Health Examination Survey (NHANES) in the United States (1999–2004), the prevalence of obesity remains lower in Lausanne[22]. The higher prevalence of overweight in men might also account for their higher prevalence of diabetes and hypertension. In clinical practice, the diagnosis of hypertension relies on several consecutive BP measurements but as in most epidemiological studies, BP measurements were conducted during a single visit [23]. Terminal digit preference in BP readings may induce a bias, a consistency check was conducted showing no significant deviation of terminal digit frequency from the 10% value for all BP measurements (not shown). The prevalence of hypertension was higher in men than in women and increased with age. Interestingly, the gender-specific prevalence rates found in the CoLaus study were similar to those previously reported for the same age group in Italy [24] and in nearby Geneva [21]. We also observed that over half of the treated hypertensive subjects had a BP ≥ 140/90 mmHg at the time of their examination, indicating the continuous need to improve treatment compliance. The prevalence of diabetes was 6.6% and was significantly higher in men and in particular after the age of 55 years. These numbers are similar to other Swiss estimates [25] and to the KORA Augsburg study in the southern part of Germany [26]. About a quarter of the CoLaus participants were smokers, a figure quite similar to the data reported for the Geneva population in 2003 [21] and somewhat lower than those observed in the USA[27]. The prevalence of smoking was higher in men than in women, although this difference tended to decrease among younger age groups, as previously reported [28]. Indeed, in the age group 45–54, the prevalence of smoking was higher in women than in men, suggesting that the "gender gap" regarding smoking no longer exists for middle-aged subjects.

The high prevalence of CVRFs in this study population underscores the necessity to increase disease awareness, to improve screening in high risk subjects and to promote prevention both at the public health and individual level.

Recently, an increasing number of reports have demonstrated the power of whole genome association studies approach in complex diseases such as diabetes [29], obesity [30] and cardiovascular disease [31]. The size of the CoLaus study, the population-based design and the in-depth phenotypisation were chosen in order to harness the power of this technology using dichotomous and continuous trait analyses. In particular, this ensures an identical phenotypisation in cases and controls, which has been a limitation in some of the recently published reports [32] and allows to study complex traits such as the metabolic syndrome. Combined analysis of our results with other datasets has already allowed to identify new genetic determinants associated with circulating LDL-plasma levels [17] (similar results were published concomitantly by other groups [33,34]) and determinants associated with height [35]. Additional analysis are currently ongoing.

### Perspectives

A more comprehensive characterization of the Colaus participants is currently ongoing. First all participants aged 35 to 65 were solicited to undergo a psychiatric investigation based on a semi-structured diagnostic interview. In addition, 500 subjects were assessed for CV functional measurements. Indeed several previous studies have revealed associations between mood disorders and, in particular depression, and CVRFs and CV diseases (reviewed in [36]). The availability of a simultaneous CV and psychiatric phenotype will allow us to further explore the epidemiologic and potentially a genetic basis for this association. Finally, 500 randomly selected non-diabetic individuals underwent a 2 hour glucose tolerance test. The results from these investigations will be reported separately.

A longitudinal follow-up of all participants in the CoLaus study is planned and shall provide essential data for trends over time of major CVRFs and on incident cases of CV diseases. In the current cross-sectional study, participants were asked whether they consented to be contacted for follow-up, with > 90% favorable responses.

## Conclusion

In summary, these initial results from the CoLaus study show that the prevalence of main CVRFs is high in the Caucasian population of Lausanne, and in particular in men. This emphasizes the need for continued epidemiological monitoring and for strengthening interventions to reduce the prevalence and severity of CVRFs in this population. This population-based study with over 6000 extensively characterized and genotyped participants constitutes a unique resource to identify new or replicate suspected or known molecular determinants of CVRFs and associated diseases.

## Abbreviations

CV: cardiovascular; CVD: cardiovascular disase; CVRF: cardiovascular risk factor; BP: blood pressure; SBP: systolic blood pressure, DBP: diastolic blood pressure; BMI: body mass index; LDL-cholesterol: low-density cholesterol; HDL-cholesterol: high density cholesterol; NHANES: National Health and Nutrition Survey; NT-proBNP: N-terminal pro-Brain Natriuretic Peptide; ASAT: aspartate aminotrasnferase; ALAT: alanine aminotransferase; Gamma-GT: gamma glutaryl transferase; CDT: carbohydrate deficient transferine; hs CRP: high sensitivity C-reactive protein. CAD: coronary artery disease, T1DM/T2DM: Type 1/Type 2 diabetes mellitus.

## Competing interests

Kijoung S. Song, Xin Yuan, Theodore M. Danoff, Heide A. Stirnadel, Dawn Waterworthand Vincent Mooser are full-time employees of GlaxoSmithkline.

## Authors' contributions

MF, VM, PV, GW, AP, DH, FP contributed to the provision of participants and study material. PV, GW, VM, DW, HS, TD, FP, DH and MP participated in the study design and conception and the coordination of the project. MF, VM, PMV, MB, KS, XY assembled the data and performed the statistical analyses. PV, MF, VM, DW, HS, PMV analyzed and interpreted the data. PV, MF, PMV drafted the article which was revised by VM, VM, MB, MP and GW. All authors read and approved the final manuscript.

## Pre-publication history

The pre-publication history for this paper can be accessed here:



## Supplementary Material

Additional file 1 Clinical chemistry and biological makers measured in the CoLaus study. Analytical procedures, maximum inter and intra-batch coefficient of variation and manufacturers for the Clinical chemistry and biological makers measured in the CoLaus study. Click here for file
