# Supplementary material for: The CoLaus study: a population-based study to investigate the epidemiology and genetic determinants of cardiovascular risk factors and metabolic syndrome
Source: BMC Cardiovasc Disord. 2008 Mar 17;8:6. doi: 10.1186/1471-2261-8-6 (PMC2311269; doi:10.1186/1471-2261-8-6)
Supplement: Additional file 1 — Clinical chemistry and biological makers measured in the CoLaus study. Analytical procedures, maximum inter and intra-batch coefficient of variation and manufacturers for the Clinical chemistry and biological makers measured in the CoLaus study. [file 1471-2261-8-6-S1.doc]

| **Marker** | **Type of assay** | **Maximum inter and intra-batch CVs** | **Manufacturer** |
| --- | --- | --- | --- |
| Adiponectin | ELISA | 8.3% - 8.3% | R&D Systems, Inc, Minneapolis, USA |
| Albumin | Bromocresol green | 2.5% – 0.4% | Roche Diagnostics, CH |
| Alanine aminotransferase (ALAT) | International Federation of Clinical Chemistry (IFCC) method at 37°C | 5.6% – 0.7% | Roche Diagnostics, CH |
| Alkaline phosphatase | International Federation of Clinical Chemistry (IFCC) method at 37°C | 3.3% – 0.7% | Roche Diagnostics, CH |
| Aspartate aminotransferase (ASAT) | International Federation of Clinical Chemistry (IFCC) method at 37°C | 3.6% – 1.8% | Roche Diagnostics, CH |
| Apolipopoprotein B (ApoB) | Turbidimetry | 8.7% - 7.6% | Polymedco, Chicago, USA |
| Calcium | O-cresolphtalein | 2.1% – 1.5% | Roche Diagnostics, CH |
| Carbohydrate deficient transferin (CDT) | separation by capillary electrophoresis (Beckman P/ACE 5510 System) using the Ceofix-CDT reagent kits (kits #10-004760) | Maximum intra-batch CV was between 3.8% (highest CDT levels) and 15.3% (lowest CDT levels) | Beckman Coulter Instruments, Switzerland  Analis, Belgium, |
| Cholesterol (Total) | CHOD-PAP | 1.6% – 1.7% | Roche Diagnostics, CH |
| Creatinine (serum and urine) | Jaffe kinetic compensated method | 2.9% – 0.7% | Roche Diagnostics, CH |
| Gamma glutamyl transferase (GGT) | Optimized standard method according to the IFCC, at 37°C | 1.6% – 0.4% | Roche Diagnostics, CH |
| Glucose | Glucose dehydrogenase | 2.1% – 1.0% | Roche Diagnostics, CH |
| HDL-cholesterol | CHOD-PAP + PEG + cyclodextrin | 3.6% – 0.9% | Roche Diagnostics, CH |
| High sensitive CRP (hsCRP) | Immunoassay and latex HS | 4.6% – 1.3% | Roche Diagnostics, CH |
| Homocystein | High pressure liquid chromatography following ammonium 7-fluorobenzo-2-oxa-1, 3-diazole -4-sulphonate (SBD-F) derivatisation | 3.1% – 2.9% | Agilent 1100 apparatus |
| Insulin | Solid-phase, two-site chemiluminescent immunometric assay | Maximum intra-assay CV of 13.7%. | Diagnostic Products Corporation, Los Angeles, USA |
| LDL particle size | Polyacrylamide gel electrophoresis, Lipoprint LDL kit®, | 1.5%-0.5% | Quantimetrix Corporation, CA, USA |
| Leptin | ELISA | 12.8% - 5.8% | American Laboratory Products Company, Windham, USA |
| NT-proBNP | ELISA |  | American Laboratory Products Company, Windham, USA |
| Total protein | Biuret | 1.3% – 0.6% | Roche Diagnostics, CH |
| Triglycerides | GPO-PAP | 2.9%-1.5% | Roche Diagnostics, CH |
| Uric acid | uricase-PAP | 1.0% – 0.5% | Roche Diagnostics, CH |

Clinical chemistry and biological makers measured in the CoLaus study with analytical procedures, maximum inter and intra-batch coefficient of variation and manufacturer
